# Proceedings of the Virginia Medical Society

**Published:** 1895-10

**Authors:** 


### Society Reports.

### PROCEEDINGS OF THE VIRGINIA MEDICAL SOCIETY.

Meeting at Wytheville, Va., September 4–7, 1895.

### PYÆMIA AND SEPTICÆMIA IN THEIR SURGICAL ASPECTS.

Honorary Fellow, DR. J. MCFADDEN GASTON, of Atlanta, Ga., read a paper on this subject. (See page 445, *et seq.*)

### STATE PROVISION FOR EPILEPTICS.

DR. WILLIAM F. DREWRY, Assistant Physician, Central Insane Hospital, of Petersburg, Va., read a paper on this subject, of which the following is an abstract:

Epilepsy is to the physician, the statistician, and the sociologist, alike, an unknown and unknowable factor. As a concomitant of idiocy, insanity and crime, it assumes enormous proportions.

The ætiology and pathology being so little understood, treatment must, necessarily, be empirical and unsatisfactory. Little can be done in the way of medical treatment except to modify the attacks or to lessen their frequency. A *cure* of epilepsy is very seldom accomplished. Certainly not more than one per cent of idiopathic cases permanently recover.

No accurate data can be procured upon which to estimate the proportion of epileptics to the general population, but two per thousand is the usually accepted ratio. In Virginia, there are probably three thousand. In the poorhouse of the State there are no less than 200, and in the insane asylums, the number of epileptics reaches 200—a total of 400 now being cared for by the State, though not in a suitable manner, for neither the almshouse nor the lunatic asylum is the proper place for them. Only the very violently insane epileptics should be kept in the hospitals for the insane. Not more than ten per cent of all epileptics become insane; many, however, deteriorate mentally, morally and physically to a marked degree, unless they are properly treated in the early stages of their diseases.

In the medico-legal aspect, there is too much difference manifested. Many crimes are closely connected with a vitiated epileptoid condition, and no one can tell when and how a sudden insane impulse may possess the erratic victim of this malady.

Owing to their peculiar disease, epileptics have great difficulty in finding employment. They are also deprived of the advantages of education, etc., of those afforded by churches and places of amusement. They are shunned and neglected. An epileptic is an unconcealed skeleton at home, an object of ceaseless anxiety. A large proportion of these afflicted families are in poverty and absorbed in struggles for a livelihood; hence, the caring for a dependent epileptic becomes a grievous and heavy burden.

For various and obvious reasons, the *State Government* is well adapted to look out for the welfare of all her delinquents and defectives, and should no longer neglect her needy epileptics. The State should care for her poor epileptics in a separate institution, suitably equipped for the treatment of their distressing malady. Protection and aid to the afflicted as well as to society in general, demand that provision of some kind be made for their care.

After years of experience and actual operation, it has become a recognized fact that special requirements of epileptics are no, where so well met as in the so-called *farm colony*. The prime objects of such a colony are to give each beneficiary the advantages of the most scientific medical treatment, education, enjoyment, the most humane custodial care, and means of regular, productive employment. To accomplish these objects, palatial structures are not required. Plain, inexpensive cottages, natural and homelike to most of the inmates, shops and other facilities for various industries, a hospital for the sick and infirm, halls or gymnasiums for recreation and amusement, chapels and schoolhouses, etc., all arranged on the village plan, and attached thereto a large farm properly equipped, to furnish employment, as well as provisions for the patients and employees, meet the requirements admirably. For those who become insane, isolated buildings of a suitable character should be provided. In such an institution—conducted on the industrial plan, the beneficiaries would not suffer the ignominy attached to the pauper class, for they would be in a degree, producers and not absolutely dependent.

The epileptic colony is not entirely a new idea, for it originated in France, and there, and in Germany, England, Sweden, Holland, Switzerland and elsewhere, it has proved to be a grand success and a source of untold good to suffering humanity.

This country has been singularly slow in recognizing her duty to thousands of epileptics within her borders, but she is beginning to be anxious in their behalf. Ohio has already a State Hospital for epileptics. New York, Massachusetts and other States are taking steps to establish colonies for them. It is the general opinion in these States that these colonies will, in a few years, become almost self-sustaining, so much work will be done by the patients. Will Virginia refuse to respond to the cry of her unfortunate epileptics? Hardly.

Our lawmakers need to be reminded of the necessity and humaneness of the special provision for epileptics. Let the medical profession, with a broad and comprehensive devotion to the general good and to individual happiness, use its best efforts in the creation of a just public sentiment in this matter.

In an article published in the *Virginia Medical Monthly* for September, 1894, the writer earnestly advocated the State care of all dependent epileptics in an institution exclusively for them. He reiterates the opinion, expressed in that paper, viz., that a farm-colony conducted on the industrial and educational plan, and provided with every facility for the most scientific medical treatment of the patients, would meet the requirements better than any other method yet suggested. It is feasible; it is economical; it is humane. It has been tried elsewhere and demonstrated beyond question to be a practical success.

Those who would reap the benefits of such an institution are:

First. The epileptics now confined in the State hospitals for the insane. By removing these—200 in number—sufficient room would be gained at these hospitals to accommodate probably all the insane of the State, in need of care and treatment, at least, for a year or two.

Second. All the epileptics now in the county and city almshouses—certainly as many as 200.

Third. Dependent epileptics; each county and city to have a number of patients proportional to its population.

Fourth. Both white and colored epileptics—separate and distinct provision being made for each race.

To determine the question of epilepsy, proper legal proceedings should be had as in the case of insanity. The State, however, is very sadly in need of a better law regulating the commitment of the insane.

In conclusion, it is not utopian to hope that in the near future, Virginia will awaken to a recognition of her duty to her epileptics, and see the wisdom of making that provision for them which is clearly demonstrated by every consideration of justice, equity and philanthropy.

### CASES OF CEREBRAL TRAUMA; INDISTINCT AND FALSE HISTORY; REMARKS ON DIAGNOSIS AND TREATMENT.

DR. M. J. PAYNE, Locust Grove, Va., forwarded the report of a case that occurred in his practice last May.

Broadus D., colored, male, age 18 years. Personal and family history good, by statement. He was first seen May 1, 1895.

His present illness came on suddenly, but he was uncertain when he first began to complain. He denied having previously consulted any physician, which was untrue.

*Nervous symptoms.*—He complained of severe headache, restlessness, and inability to sleep, and appeared excited and very nervous; had an empty, half-gazing stare, with red and suffused eyes; no photophobia; vision normal; pupils reacted normally. He would now and then quickly spring up and suddenly dart across the room if not detained. This excitement approached very nearly to maniacal.

*Digestive symptoms.*—Anorexia, some little thirst, and almost absolute constipation. Tongue coated heavy and pasty white; breath heavy. No tympany, no tenderness, no iliac gurgling. Just above the pubes, he stated there was a severe pain, but no tenderness; no cystitis. Had never had specific trouble. Functions of bladder normal. Urine rather scant, otherwise normal. Pulse, 72—slow, full, and regular. Heart and lungs normal. Temperature, 102.8 degrees. Skin, hot and dry. He was carefully questioned in regard to having been struck on the head or having fallen in wrestling, or from a wagon, or having been thrown from a horse, etc. This he and his parents positively denied.

May 2. Pulse, 62; temperature, 102 degrees. General condition about the same, though more quiet; complained that he could not see clock face near by.

May 3. Pulse, 64; temperature, 102 degrees. Apparently better; slept well; not so restless as on day previous; vision much more affected.

May 4. Pulse, 62; temperature, 103.2 degrees. Stupid, dull, apathetic; answered questions slowly and hesitatingly, and often incorrectly—muttering some delirious reply, such as "Old Isaac says I can't drag the plow up the hill backwards and chop it up fine." "Me and Ed are going to—" then a low muttering and a restless shuffle of position. On arousing him, he would get up and know physicians and others, but would lapse at once into stupor and delirium. Vision distinctly affected; no photophobia; said his head did not ache.

May 5. Pulse, 67; temperature 103.2 degrees; very dull and delirious, but somewhat better; hair was clipped short and ice applied to scalp.

May 6. Pulse, 70; temperature, 103.2 degrees; stupor deepening, approaching coma; could, however, be aroused, and would do as told, hesitatingly and very slowly, often stopping for a moment, then continuing. Digestive symptoms much better; pain over pubes disappeared, and no headache

May 7. Pulse, 85; temperature, 103.2 degrees; comatose, deeply unconcious, insensible, skin hot and dry, constipation absolute; would not protrude tongue; could, however, swallow very well. There was right hemiplegia, with no loss of sensation, pupils immobile, did not react at all; both pupils dilated, but left pupil widely dilated. Tendency to right internal strabismus with some nystagmus of right eye; left eye fixed, looking steadily to the front; eyelids half open; respirations sighing and irregular; no stertor.

May 8. Pulse, 90, and weak; coma deep. Symptoms same as day before; in addition face was involved in paralysis; scalp was shaved, and on examination a marked localized œdema surrounding the left parietal boss was noticed. Temperature left side, 102.2 degrees; right side, 103 degrees. As on May 1, a careful examination failed to reveal any fracture or depression, or ridge of skull; no cracked-pot sound, but pain and a peculiar movement of left leg and arm were elicited by percussion. A number of small scars were noticed, however—one sore not entirely healed. This irregular appearance caused me to think of the uneven surface of the ground. I afterwards learned from "outside talk" that he had been thrown from a colt some six weeks before being taken ill, and had spent Easter Monday night in a drunken carousal with some friends in the woods.

*Diagnosis.*—Where an injury is so positively denied, however strong the suspicion may be, greater care has to be exercised in arriving at a correct opinion of the cause. It will be remembered that a history of an injury could not be obtained in this case; yet there existed a strong suspicion in my mind, that the parents knew more than they had told. So often do we notice little betrayals in the actions and habits of our patients, which, while not conclusive, often turn suspicion into the right path, and thus a correct opinion is formed without a true and full history.

Acute traumatic encephalitis was the diagnosis then, because "the injury" was suspicioned and the suspicion was confirmed by the course of the case. Acute traumatic encephalitis begins in from one to three days after the injury, and gives rise to headache, pain and elevation of surface temperature, at the seat of injury (to the brain substance), restlessness, insomnia, excitement, delirium, general fever, and full and frequent pulse; as compression comes on, the pulse becomes slower; constipation and probably vomiting occur. As the disease advances, twitching of the muscles, convulsions, stupor, deepening into absolute coma, and paralysis ensue in successive order. A frequent and irregular pulse, clammy sweating, and dilated pupils argue unfavorably. The paralysis is due to compression brought about by inflammatory exudation.

Chronic traumatic encephalitis presents somewhat the same picture as acute encephalitis, differing in that, in the chronic form, the onset is several weeks or months later, is more insidious and the symptoms are not so pronounced. (In this case, however, the patient stated that the onset was sudden, and the symptoms were very pronounced.) Mental hebetude, epileptiform convulsions, and irritable temper are early noticed. Choked disk, with no loss of acuteness of vision, will be found on an ophalmoscopic examination. Later, localized paresis or paralysis, followed by coma and death due to the formation of an abscess unless relief is given by removal of the pus. While it is usual for photophobia and intolerance to sound to exist in acute encephalitis, yet an absence of these almost constant symptoms was noticed in this case. I believe that the marked failure of vision in this case was due to œdema of the retina.

*Prognosis.*—Acute traumatic encephalitis, as a rule, speedily terminates in death. The chronic form gives a better opportunity for suitable treatment and, therefore, recovery, though often it, too, is fatal.

*Treatment.*—The main indication was to lessen or control the inflammatory condition of the brain and its membrane. The treatment was as follows: Rest in bed in a darkened room and careful watch kept. Warm baths and extreme cleanliness. Milk diet. Purgatives, such as calomel, jalap and salines were administered with the idea of lowering the intracranial pressure and controlling inflammation. They all were very inefficient. Enema of hot water had to be resorted to in addition. Often purgatives fail to act when there is marked intracranial pressure. Often in apoplexy, purgatives will fail to act; if you bleed even but a little, the purgatives then act severely. The bromides, two drachms per diem, were used without effect. Cannabis indica (fluid extract), for insomnia and restlessness, acted well. Mercury and opium were given to combat the inflammation without any beneficial influence whatever. Ice to the head and sinapisms to the feet proved valueless. In short, the case progressed from bad to worse.

On May 9th, the localizing symptoms having become sufficient to locate the lesion, with the help of Dr. James M. Scott, an operation was done.

In chronic encephalitis, the usefulness of operative interference is clearly established. In the brain, pus and inflammatory products cannot gain a natural outlet. The first rule in surgery is to "Let out the pus" (Keen). Pus confined in the brain should be let out. While it is considered bold to operate in acute traumatic encephalitis, and there are not a sufficient number of cases on record to judge of its usefulness, yet the inflammatory products should be removed just so soon as the trouble can be located. This is imperative. Indeed, if the symptoms of pressure be very pronounced, a simple trephine operation, done early, will be of great benefit. If there exists a scar, make this the site of the operation; and if the dura bulges and is discolored, open it and see the nature of the trouble. Your patient will probably die anyhow; therefore give him the best possible chance. (This is before the occurrence of localizing symptoms.)

*Location of the lesion.*—In this case, right hemiplegia, with facial paralysis following, later aphasia, and third, nerve paralysis, showing very plainly that the lesion was located on the left side, and that the ascending frontal and ascending parietal, with Broca's convolution, were affected. The course of the paralysis, which was first leg, then arm, then face and oculo-motor nerve, and later aphasia, showed that the lesion started above and progressively extended downward. It is possible that the distinct affection of the vision might have been due to pressure of the cuneus, but I could not gain clear enough answer from the patient to prove this, and hence it could have been due to œdema of retina.

*Operation.*—The fissure of Rolando was marked out and the scalp turned back, using the large horseshoe shaped flap. The trephine was applied over the center for the leg, and on removing the button of bone, the dura bulged into the opening. No pulsation of the brain was detected. The dura was then opened, and the arachnoid and pia were found to be dark, congested and breaking down. Lying on the brain surface was a dark, organized inflammatory exudate, which was beginning to undergo softening. The trephine was again applied lower down, after enlarging the opening and gaining more room to work. The same condition of affairs was noticed. The general circulation of the patient was noticed to improve distinctly. Attention was now directed to the removal of all of the exudate, but, as we advanced, it was found that the grey matter on the surface of the convolutions was undergoing rapid inflammatory changes. It therefore would have been foolish to have tried further to remove any of the inflammatory product, for the extent and depth of the lesion were beyond the pale of the knife. This, then, was necessarily a fatal case. The patient lived about six hours after the operation.

I was surprised when he was placed back on the bed to see the pulse and respiration grow better. A pulse of 140 came down to 128. Respiration now quiet and regular. Unfortunately, no post-mortem could be had.

Dr. Payne reported the case for the following reasons:

1st. It is not often that a local surgeon or physician is called upon to treat a case of acute traumatic encephalitis.

2d. To show that, while one case of acute encephalitis (American Text-book of Surgery) was benefited by operative interference, yet all cases are not amenable to this method of treatment.

3d. To show the uselessness of medical treatment, and to express a positive conviction that the best possible success is promised in early and wise operative interference.

4th. To show the absolute necessity of a minute knowledge of the technique of cerebral surgery and cerebral topography.

In the discussion of DR. GEORGE BEN. JOHNSTON'S paper, on the treatment of Cholelithiasis, DR. J. MCFADDEN GASTON, of Atlanta, remarked that while the reader claimed only to present some points for discussion, he had occupied most of the ground ordinarily taken up by those familiar with gall bladder surgery. There were, however, some matters not usually dwelt upon by writers, to which attention might be directed. The first and most important consideration pertains to the treatment of cases in which there are gallstones in the gall bladder and ducts, which call for removal by incision, and it is found impracticable to attach the sac of the gall bladder to the skin or the intestines, is to suture the wall of the duct. In these cases, it has been proposed to tampon the intervening space to the external opening with gauze, and leaving the ends of the strips protruding, drainage is accomplished, and the bile or other discharge is kept from entering the peritoneal cavity. While fresh bile is not found to be an irritant to the peritoneum, the vitiated discharge which takes place after obstruction proves hurtful, and should not be allowed to enter the abdominal cavity.

Dr. W. E. B. Davis has taken a prominent part in urging this mode of procedure, and has made numerous experiments upon infirm animals, illustrating the advantage of introducing iodoform gauze into the wound for the purpose of walling off the intestines and other viscera, while it serves, by capillary attraction, to carry off the discharge.

This has been practiced by others with the best results; and to Dr. Davis is due the credit of impressing upon the profession the advantages of this treatment in cases not admitting of the attachment of the gall bladder to the external opening or the intestines, and in which it is found impracticable to evenly suture an incision of the common duct after the removal of biliary calculi.

Attention may also be drawn to the applicability of this process to cases of rupture of the gall bladder, either by traumatism, or as a result of disease.

In an extremely aggravated case of collapse from rupture of the gall bladder, which came under my observation in consultation with two colleagues in Birmingham, Ala., peritonitis had already been developed, with profound vital prostration. Although we all concurred in the desperate character of the case as offering no prospect of relief, yet death being certain without an operation, it was determined to give the patient the only chance for escape. An oblique incision in the right side below the points of the ribs reached a ruptured gall bladder with general functions. The cavity was thoroughly injected with hot borated solution, and the wound filled with iodoform gauze, leaving the ends out at the external end. The patient did not rally, and sank within four hours. If this course had been adopted very soon after the rupture, it is reasonable to infer that she might have recovered.

Another case has come under my observation in a lady who had suffered for months with hepatic disorders, and finally presented localized pain and distension in the region of the gall bladder, which was seen in consultation, after aggravated symptoms with peritonitis were developed. Being convinced that there were adhesions around the circumscribed inflammation, which shut off the gall bladder from the abdominal cavity, my advice was given to treat the case upon general principles, notwithstanding the indications pointed to a ruptured gall bladder.

Small doses of calomel and soda, followed by Epsom salts, with some tea, were given daily, so as to keep up free purgation; and a local application was made twice a day of equal parts of the ointment of mercury, comp. iodine camph., and belladonna. These, with other less important remedies, secured a favorable result, and the general health of the patient has been good since.

The members of this society may be interested in some details of a case of profound cholæmia with galls, in which an operation was done some months ago, and no report has ever been made to the profession.

Upon cutting down upon the gall bladder, just beneath the border of an enormously enlarged liver, the sac was found closely grasping a mass of gallstones, and others occupied the cystic, and common ducts, so that the angularity of the ducts was almost obliterated entirely. After incising the contracted gall bladder and extracting, with scoops and forceps, twelve large biliary calculi from the sac and ducts, it was found that there was an adhesion of the walls of the common ducts and the colon. An incision through this would have established a communication with the gall bladder and intestines; but as there was no trace of bile present to flow out, the only result would have been the entrance of fæcal matter into the duct, so that any such a procedure would have done harm. The common duct, below the position occupied by the calculi, was entirely occluded, and, so far as could be ascertained, the hepatic ducts above were agglutinated. The atrophied gall bladder and enlarged cystic and common ducts were packed with iodoform gauze, and the space around them was stuffed with the same material, having the ends of the strips protruding from the external wound, for the purpose of drainage. This was removed on the third day, and was followed by a profuse discharge of dirty sanious fluid, which indicated that there was a breaking down of the structure of the liver. On the next day there was some sign of bile in the discharge, leading to the conclusion that ulceration of the hepatic ducts had taken place. Subsequently there was a copious flow of bile from the wound, and a drainage tube was kept in to carry it off, but more escaped around the outside of the tube than through it, and excoriated the skin and the opening. The enlargement of the liver was only slightly diminished, and the jaundice persisted; yet, for a week, matters looked promising, notwithstanding the marked vital depression. Bile continued to flow from the wound in great abundance, until the end of the second week, when it gradually diminished. She was sustained by milk punch and beef soup, using an alternative tonic of Huxham's tinct. cinchona and lupulin with nux vomica and chlorate potash, in tablespoonful doses every three hours. There was marked lowering of temperature—below the normal at times—when subcutaneous injections of strychnine and nitro glycerin were used. This occurred especially in the morning and evening. All, however, was unavailing, and, at the end of three weeks, the patient died, from the effects of the cholæmia.

My impression is, that the occlusion of the hepatic ducts gave way under a process of ulceration, and discharged outside of the common duct. The enormous size of liver prevented a proper examination of the hepatic duct at the time of the operation, and, could the exact state of things have been known, force might have been used to open the hepatic duct, and thus give an outlet into the natural channel.

One other point in connection with cholelithiasis is worthy of note, looking to the removal of gallstones without any operative measures. It is known that biliary calculi are sometimes discharged after an attack of hepatic colic by the dislodgment of the stone from the common duct, and I have become satisfied that calculi are capable of escaping from the gall bladder in some cases under the influence of medication. It is not supposed that they undergo any change in their structure, but under the relaxing and lubricating effect of olive oil, there is abundant evidence in the reports which have been made by observing that the discharge of gallstones follows the free use of olive oil. My individual experience confirms what is claimed for this remedy, and in doses of four ounces every three hours until twelve or sixteen ounces are taken, in a case with gallstones, I have seen them discharged with the fæcal evacuations.

Some have fallen into an error in supposing that the spherical masses of fatty matters and cholesterin which generally appear in the evacuations after taking olive oil, are gallstones. It may be that calculi will be contained in these balls or mixed with them, but nothing can be inferred from these cholesterin formations in regard to the presence of gallstones. These are often of considerable firmness, but will break under moderate pressure between the thumb and forefinger, whereas a biliary calculus resists pressure, and generally the surface has facts showing that it has been rubbing against other stones. Even when gallstones are not found after giving the olive oil, there is frequently relief to the attacks of hepatic colic, and patient has been spared the ordeal of a surgical operation. It is, therefore, regarded as commendable practice in suspected cases.

In regard to the history of gall bladder surgery, I have to state, that the first cholecystotomy was performed by Bobbs, and the first cholecystenterostomy was done by Minnewater, when my experiments on dogs were undertaken, supplemented by those of other experimenters in this field, and awakened a new interest in this department of surgery. Quite a large number of cases of cholecystotomy, cholecystectomy, and cholecystenterostomy, with a few cases of choledectomy, have been reported by operators in this country and in other parts of the world, and the surgery of the gall bladder is now fully recognized by the profession.

In reply to a question from DR. MOORE, of Richmond, as to his view of function of the gall bladder, DR. GASTON said, that he accepted the position of some, that it was a diverticulum or reservoir for the bile, which was expelled gradually, to be mixed with the chyme as it passed through the duodenum. That this process depended upon the contractility of its coats in part, and the distended stomach exerted pressure upon it also. The bile was conducive to intestinal digestion, and promoted peristalsis, while it forces regularity in the fæcal evacuations, so that its loss is detrimental.

